# Preparation and near-infrared photothermal conversion property of cesium tungsten oxide nanoparticles

**DOI:** 10.1186/1556-276X-8-57

**Published:** 2013-02-05

**Authors:** Cheng-Jia Chen, Dong-Hwang Chen

**Affiliations:** 1 Department of Chemical Engineering, National Cheng Kung University, Tainan, 701, Taiwan

**Keywords:** Cesium tungsten oxide, Nanoparticles, Near infrared, Photothermal conversion, Bead milling

## Abstract

Cs_0.33_WO_3_ nanoparticles have been prepared successfully by a stirred bead milling process. By grinding micro-sized coarse powder with grinding beads of 50 μm in diameter, the mean hydrodynamic diameter of Cs_0.33_WO_3_ powder could be reduced to about 50 nm in 3 h, and a stable aqueous dispersion could be obtained at pH 8 via electrostatic repulsion mechanism. After grinding, the resulting Cs_0.33_WO_3_ nanoparticles retained the hexagonal structure and had no significant contaminants from grinding beads. Furthermore, they exhibited a strong characteristic absorption and an excellent photothermal conversion property in the near-infrared (NIR) region, owing to the free electrons or polarons. Also, the NIR absorption and photothermal conversion property became more significant with decreasing particle size or increasing particle concentration. When the concentration of Cs_0.33_WO_3_ nanoparticles was 0.08 wt.%, the solution temperature had a significant increase of above 30°C in 10 min under NIR irradiation (808 nm, 2.47 W/cm^2^). In addition, they had a photothermal conversion efficiency of about 73% and possessed excellent photothermal stability. Such an effective NIR absorption and photothermal conversion nanomaterial not only was useful in the NIR shielding, but also might find great potential in biomedical application.

## Background

Plasmonic nanomaterials could exhibit special absorption via the excitation of surface plasmon [1-3], and the maximum absorption band was highly sensitive to the particle’s size [4,5], shape [6], local environment [7], and the coupling between near nanoparticles [8]. Furthermore, under optical illumination, they could convert the absorbed photon energy into heat energy in approximately 1 ps and then transfer the heat to the surrounding media in tens of picoseconds [2-4,9]. Such an efficient light-to-heat conversion property made them become useful as nanoheaters and therefore gain more and more attention in the past decade [1,9].

Photothermal therapy is an attractive therapy technique using photosensitizers to generate heat from light absorption and then kill the cancer cells [10,11]. To avoid the nonspecific heating of healthy cells and allow deeper penetration into tissues, near-infrared (NIR) light is usually utilized [12]. Furthermore, because the use of plasmonic nanomaterials as photosensitizers makes this technique possess spatial selectivity, a lot of plasmonic nanomaterials with NIR photothermal conversion property have been examined. Typical examples include gold nanorods [13-15], gold nanoshells [16,17], gold nanocages [18], single-walled [19-21] or multi-walled [22] carbon nanotubes, graphene or reduced graphene oxide [23], and germanium [24]. Among them, gold-based nanomaterials received the most attention, owing to their good biocompatibility and tunable optical property. However, gold is an expensive noble metal, and the preparation of its nanostructures with NIR photothermal conversion property usually needs an accurate synthesis condition or repeated deposition. Thus, the alternatives with lower cost or simpler preparation method are still in demand [25].

Recently, to reduce the energy consumption for air-conditioning and decrease the emission of carbon dioxide, NIR-shielding materials have received considerable attention in the development of transparent and solar heat-shielding filters for solar control windows of automobiles and architectures [26-34]. Among various materials with the capability of shielding NIR light via reflection or absorption mechanism, cesium tungsten oxide (particularly Cs_0.33_WO_3_) nanoparticles have been regarded to be highly promising in transparent solar filter application [26-30]. Because of the strong absorption in the NIR region, owing to the free electrons or polars, they also might be efficient as a photosensitizer in NIR photothermal therapy. However, their utilization in heating the reaction media or photothermal therapy via NIR photothermal conversion has not been reported.

Until now, only limited work has been reported for the solvothermal synthesis of cesium tungsten oxide nanorods [27]. The main method for the synthesis of cesium tungsten oxides was the solid state reaction [28]. To obtain the nanosized powder, further grinding was necessary. Thus, in this work, Cs_0.33_WO_3_ nanoparticles were prepared by a stirred bead milling process. Although Takeda and Adachi have reported the preparation of tungsten oxide nanoparticles by milling in organic medium with a dispersant agent [28], for future possible biomedical application and avoiding the use of toxic organic solvent, an aqueous milling process of Cs_0.33_WO_3_ nanoparticles without extra dispersant agents which have not been reported was attempted in this work. The appropriate pH of dispersion solution for grinding was determined, and the effect of grinding time on the size of Cs_0.33_WO_3_ nanoparticles was examined. Furthermore, the NIR photothermal conversion property of the resulting Cs_0.33_WO_3_ powder after grinding for various times was studied to demonstrate the excellent NIR photothermal conversion property of Cs_0.33_WO_3_ nanoparticles.

## Methods

Cesium tungsten oxide (Cs_0.33_WO_3_) coarse powder with a primary particle size of about 1 to 2 μm were obtained from the Industrial Technology Research Institute of Taiwan (ITRI). Deionized water was produced by Direct-Q3 ultrapure water system of Millipore Co., Billerica, MA, USA. Potassium hydroxide was purchased from Wako Pure Chemical Industry Co., Ltd (Osaka, Japan). Nitric acid was supplied by Merck KGaA (Darmstadt, Germany). The yttrium-stabilized zirconia (95% ZrO_2_, 5% Y_2_O_3_; density 6,060 kg/m^3^) grinding beads with a diameter of 50 μm were obtained from Toray Ind., Inc. (Tokyo, Japan). Polyethylene glycol 6000 (PEG 6000; molecular weight 7,000 to approximately 9,000 daltons) was a product of Merck KGaA.

Cs_0.33_WO_3_ nanoparticles were prepared via a stirred bead milling process using high-performance batch-type stirred bead mill JBM-B035 manufactured by Just Nanotech Co., Ltd, Tainan, Taiwan. This mill consists of a rotor, a mill chamber, and grinding beads. The rotor and mill chamber are made of highly wear-resistant materials: sintered silicon carbide. The mill chamber is cooled with water and has a net grinding charmer volume of 350 mL. The grinding beads are fluidized by the rotor in the mill chamber as the grinding medium. For the typical stirred bead milling process, Cs_0.33_WO_3_ coarse powder (10 wt.%) was added to the aqueous solution of potassium hydroxide at pH 8, and then the dispersion was put into the stirred bead mill. An agitation speed of 2,400 rpm (peripheral speed 10 m/s) was used to exert both shearing and imparting forces on the Cs_0.33_WO_3_ coarse powder and was run for different times. Samples were taken at various intervals of grinding time for particle size analysis. The filling ratio of the mill chambers by grinding beads was 60 vol.%. The mill was operated at a constant temperature of 20°C.

The zeta potential and mean hydrodynamic diameter of Cs_0.33_WO_3_ nanoparticles in the aqueous dispersion were measured using a Malvern Nano-ZS dynamic light-scattering spectrometer (Malvern Instruments Ltd., Worcestershire, UK). For the measurement of zeta potential, the concentration of Cs_0.33_WO_3_ nanoparticles was 10 mg/L, and the pH of aqueous dispersion was adjusted by the addition of potassium hydroxide or nitric acid. Transmission electron microscopy (TEM) analysis was carried out on a Hitachi model H-7500 (Hitachi High-Tech, Minato-ku, Tokyo, Japan) at 120 kV. High-resolution TEM (HRTEM) image of a single Cs_0.33_WO_3_ nanoparticle and the corresponding electron diffraction pattern were observed using a Jeol model JEM-2100F (JEOL Ltd., Akishima, Tokyo, Japan) at 200 kV. The content of the contaminant ZrO_2_ from the stirred bead milling process was determined using an energy dispersive X-ray (EDX) spectrometer attached to the TEM. The crystal structure was characterized by X-ray diffraction (XRD) analysis on a Shimadzu RX-III X-ray diffractometer (Shimadzu Corporation, Nakagyo-ku, Kyoto, Japan) using CuK*α* radiation (*λ* = 0.1542 nm). The absorption spectra were measured by a Jasco V-570 UV–vis-NIR spectrophotometer (Jasco Analytical Instruments, Eaton, MD, USA).

The NIR photothermal conversion property of Cs_0.33_WO_3_ nanoparticles was investigated in deionized water at different concentrations. The aqueous dispersion of Cs_0.33_WO_3_ nanoparticles was added to a 2-mL polystyrene cell, and then the dispersion was exposed to an 808-nm diode laser (HPM (LD1202) X26, Power Technology Inc., Little Rock, AR, USA) with an irradiation area of 0.3 cm^2^ and an intensity of 820 mW (i.e., 2.73 W/cm^2^). The temperature of aqueous dispersion was detected with a thermocouple. Photothermal conversion efficiency was calculated using the method reported by Chen et al. [35]. For the study on the photothermal stability of Cs_0.33_WO_3_ nanoparticles under NIR irradiation, the aqueous dispersion of Cs_0.33_WO_3_ nanoparticles (0.08 wt.%, obtained after grinding for 3 h) was continuously re-exposed to an 808-nm diode laser (2.73 W/cm^2^) for 5 cycles. For each cycle, the aqueous dispersion was irradiated for 10 min and then cooled to the initial temperature. Using a thermocouple, the variation of temperature with time was monitored.

## Results and discussion

**Figure 1:**
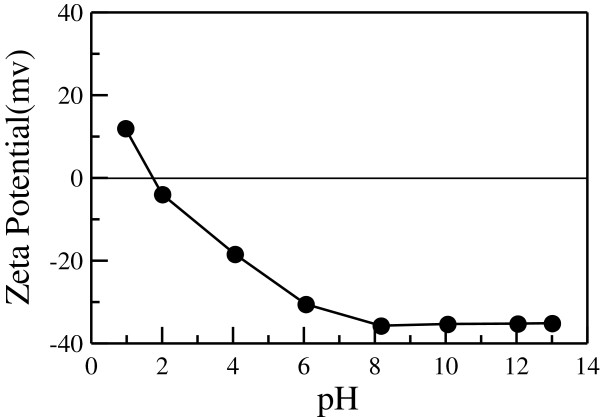
**Effect of pH on the zeta potential of Cs**_**0.33**_**WO**_**3 **_**nanoparticles in aqueous solutions.**

In this work, the bead milling of Cs_0.33_WO_3_ coarse powder was performed in aqueous solution in the absence of extra stabilizers. The resulting Cs_0.33_WO_3_ nanoparticles were stabilized in aqueous solution via electrostatic repulsion mechanism, owing to their electric double layer. Since the electrostatic repulsion was strongly influenced by the surface charge of particles, the effect of pH on the zeta potential of Cs_0.33_WO_3_ nanoparticles was investigated to determine the appropriate solution pH. As indicated in Figure 1, the preliminary study revealed that Cs_0.33_WO_3_ nanoparticles had an isoelectric point of about pH 1.8. With increasing pH, their zeta potential decreased and then approached a constant of about −35 mV when pH was above 8. Thus, the aqueous solution for the bead milling of Cs_0.33_WO_3_ coarse powder was fixed at pH 8 by adding potassium hydroxide in deionized water.

**Figure 2:**
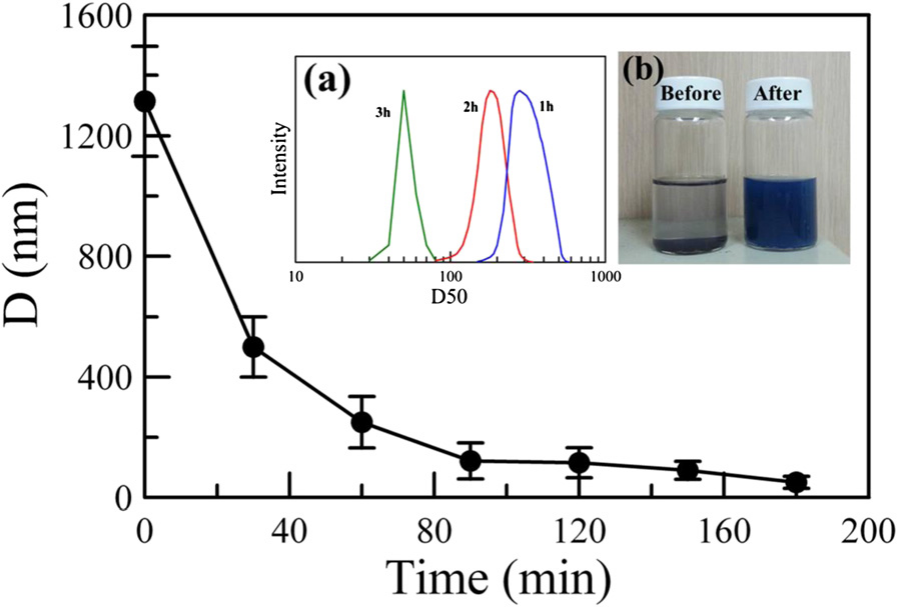
**Variation of mean hydrodynamic diameter of Cs**_**0.33**_**WO**_**3 **_**powder with grinding time.** Inset a indicates the hydrodynamic diameter distributions of Cs_0.33_WO_3_ powder after grinding for 1, 2, and 3 h. Inset b shows the photographs for the aqueous dispersions of Cs_0.33_WO_3_ powder before and after grinding for 3 h.

Figure 2 shows the variation of mean hydrodynamic diameter of Cs_0.33_WO_3_ powder with grinding time. It was obvious that the mean hydrodynamic diameter of Cs_0.33_WO_3_ powder decreased quickly from about 1,310 nm to about 50 nm within 3 h, revealing that the size of Cs_0.33_WO_3_ powder could be reduced to nanoscale efficiently by the bead milling process. Inset a in Figure 2 indicates the hydrodynamic diameter distributions of Cs_0.33_WO_3_ powder after grinding for 1, 2, and 3 h. It revealed that increasing the grinding time not only led to the decrease of hydrodynamic diameters, but also made the hydrodynamic diameter distribution become narrower. This could be reasonably referred to the fact that a longer grinding time could provide more contact between the grinding beads and the ground powder. In addition, inset b in Figure 2 shows the photographs for the aqueous dispersions of Cs_0.33_WO_3_ powder before and after grinding for 3 h. It was observed clearly that the aqueous dispersion of Cs_0.33_WO_3_ powder before grinding was quite unstable. They precipitated completely in a few minutes. However, after grinding for 3 h, a homogeneous and stable aqueous dispersion of Cs_0.33_WO_3_ nanoparticles with a mean hydrodynamic diameter of 50 nm could be obtained.

**Figure 3:**
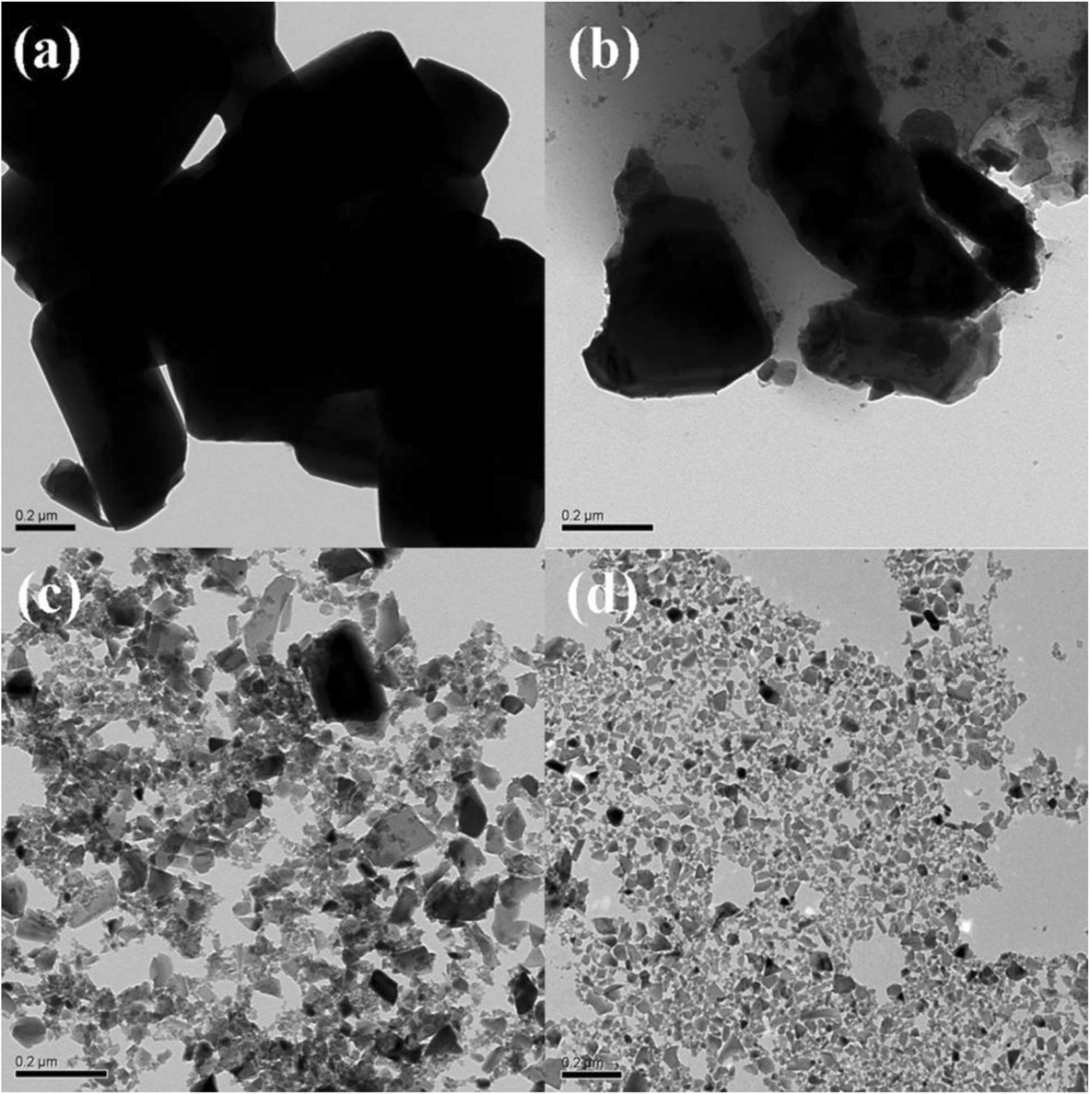
**Typical TEM images of the Cs**_**0.33**_**WO**_**3 **_**powder.** These images are before grinding (**a**) and after grinding for 1 (**b**), 2 (**c**), and 3 h (**d**).

Typical TEM images of the Cs_0.33_WO_3_ powder before grinding and after grinding for different times were shown in Figure 3. It was obvious that the Cs_0.33_WO_3_ powder before grinding had a large particle size. After grinding, the resulting particles had an irregular shape because they were debris from the collisions with grinding beads during the milling process. Furthermore, with increasing the grinding time, the particle size became smaller and more uniform. This result was consistent with the abovementioned observation of hydrodynamic diameter and confirmed that the Cs_0.33_WO_3_ nanoparticles with uniform size could be obtained by a stirred bead milling process.

**Figure 4:**
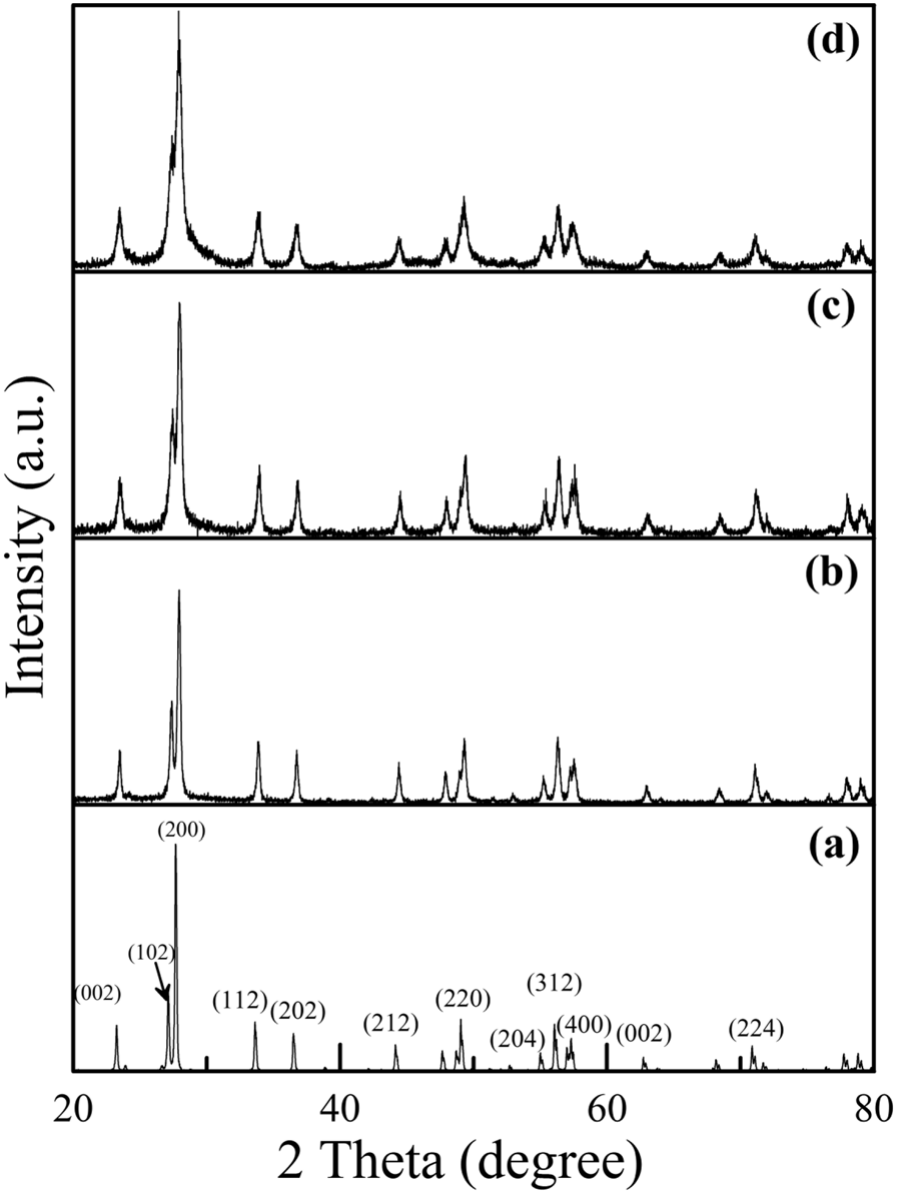
**XRD patterns of the Cs**_**0.33**_**WO**_**3 **_**powder.** These patterns are before grinding (**a**) and after grinding for 1 (**b**), 2 (**c**), and 3 h (**d**).

Figure 4 shows the XRD patterns of the Cs_0.33_WO_3_ powder before grinding and after grinding for different times. It was found that, before grinding, the characteristic peaks of Cs_0.33_WO_3_ powder corresponding to the (002), (200), (112), (202), (212), (220), (204), (312), (400), and (224) planes of hexagonal structure as indicated in the JCPDS file (PCPDFWIN v.2.02, PDF no. 831334) were observed. After grinding, the XRD patterns had no significant change except that the characteristic peaks became broader. This revealed that the bead milling process did not result in the crystal structure change of Cs_0.33_WO_3_ nanoparticles. As for the broader characteristic, it was due to the decrease in particle size. In addition, it was mentionable that ZrO_2_ might be present in the Cs_0.33_WO_3_ nanoparticles as a contaminant generally because the grinding beads might be crushed during the stirred bead milling process. However, no significant characteristic peaks for monoclinic and cubic ZrO_2_ were observed in Figure 4. This might be due to the much lower hardness of Cs_0.33_WO_3_ powder than the yttrium-stabilized zirconia grinding beads; thus, it revealed that the contamination from grinding beads could be neglected.

**Figure 5:**
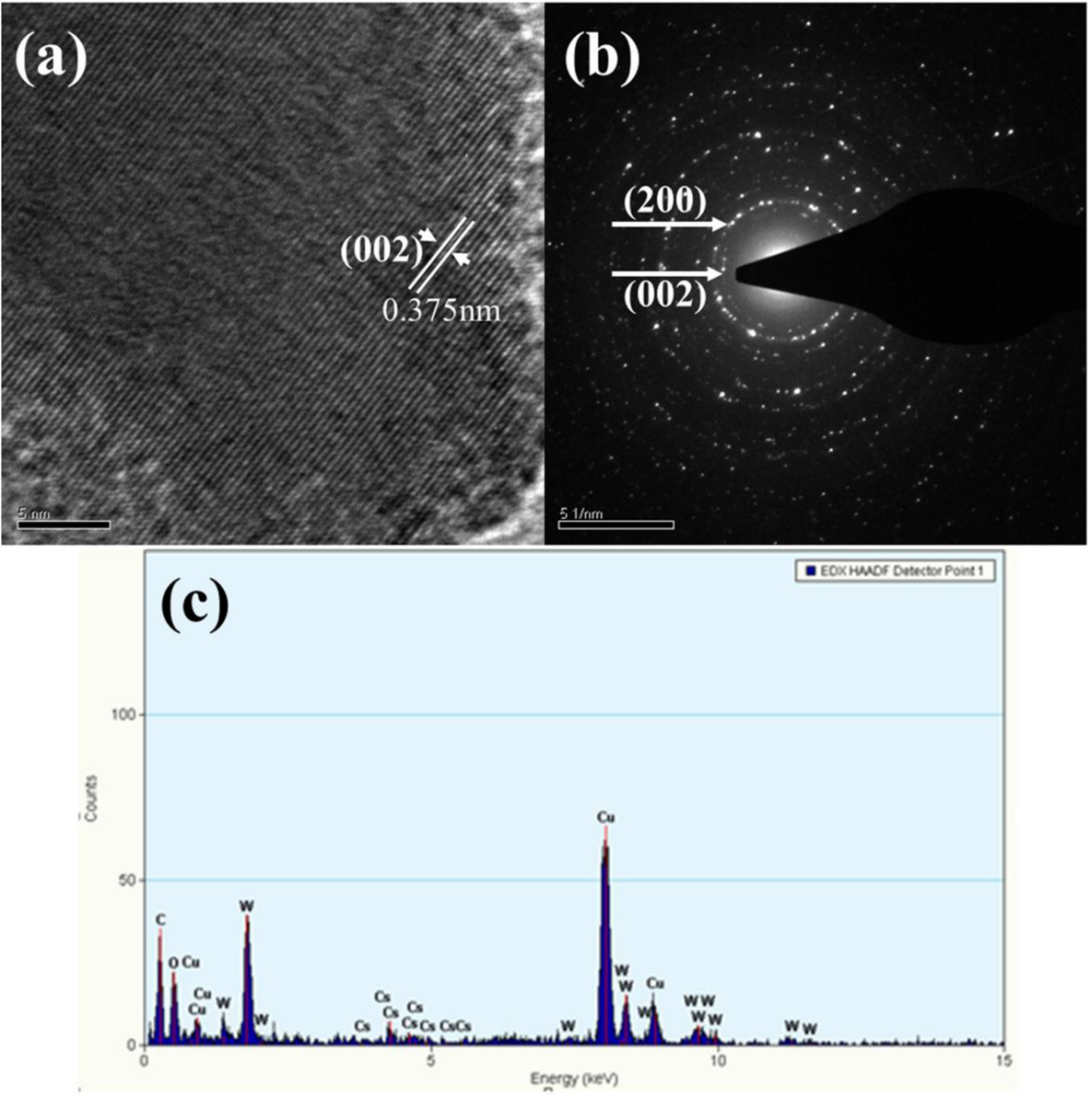
**HRTEM image (a), electron diffraction pattern (b), and EDX spectrum (c) of typical Cs**_**0.33**_**WO**_**3 **_**nanoparticle.**

Figure 5a shows the HRTEM image of a typical Cs_0.33_WO_3_ nanoparticle obtained after grinding for 3 h. The main lattice spacing of 0.375 nm is related to the (002) planes of hexagonal structure. The corresponding electron diffraction pattern was indicated in Figure 5b. Two main fringe patterns with plane distances of 3.25 and 3.71 Å could be observed. They were attributed to the (200) and (002) planes of hexagonal Cs_0.33_WO_3_. In addition, the EDX spectrum was also shown in Figure 5c. Except for C and Cu elements from the Formvar-covered copper grid, only Cs, W, and O elements were observed. No significant peak for the Zr element was found, confirming that the contamination from grinding beads could be neglected.

**Figure 6:**
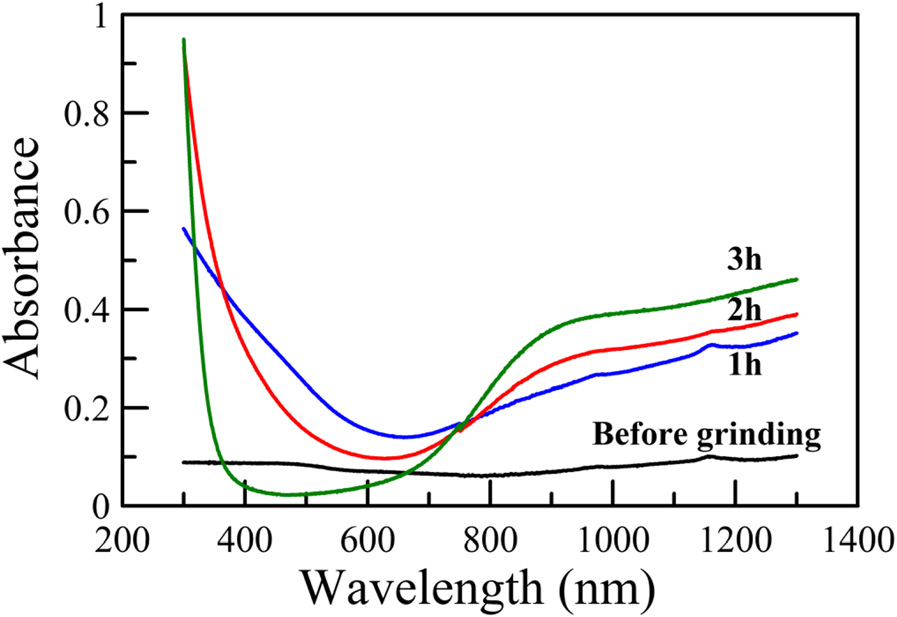
**Absorption spectra for aqueous dispersions of Cs**_**0.33**_**WO**_**3 **_**powder (0.008 wt.%) before and after grinding for different times.** For the samples before and after grinding for 1 and 2 h, 5 wt.% of PEG 6000 was added.

**Figure 7:**
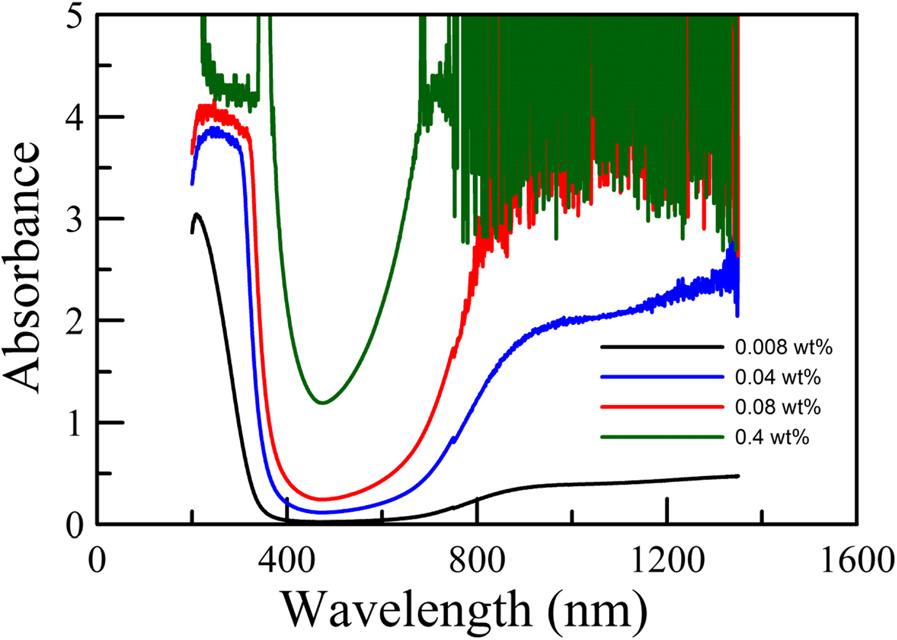
**Absorption spectra for aqueous dispersions of Cs**_**0.33**_**WO**_**3 **_**nanoparticles with different particle concentrations obtained after 3-h grinding.**

The absorption spectra for the aqueous dispersions of Cs_0.33_WO_3_ powders (0.008 wt.%) before and after grinding for different times were indicated in Figure 6. For the samples before grinding and after grinding for 1 and 2 h, 5 wt.% of PEG 6000 was added to avoid the occurrence of precipitation during the measurement. It was found that Cs_0.33_WO_3_ powder had no significant absorption before grinding. However, after grinding, the Cs_0.33_WO_3_ nanoparticles exhibited a significant absorption in the NIR region, owing to the free electrons or polarons as discussed in the work of Takeda and Adachi [28]. Also, with increasing grinding time, the NIR absorption became more significant while the visible absorption decreased. This revealed that the size reduction to nanoscale indeed made Cs_0.33_WO_3_ powder become efficient as a transparent NIR absorption material. In addition, Figure 7 shows absorption spectra for the aqueous dispersions of Cs_0.33_WO_3_ nanoparticles with different particle concentrations obtained after grinding for 3 h. It was obvious that NIR absorption could be enhanced by increasing particle concentration. When the particle concentration was above 0.08 wt.%, the fluctuation of absorbance due to the strong absorption has reached the instrumental detection limit.

**Figure 8:**
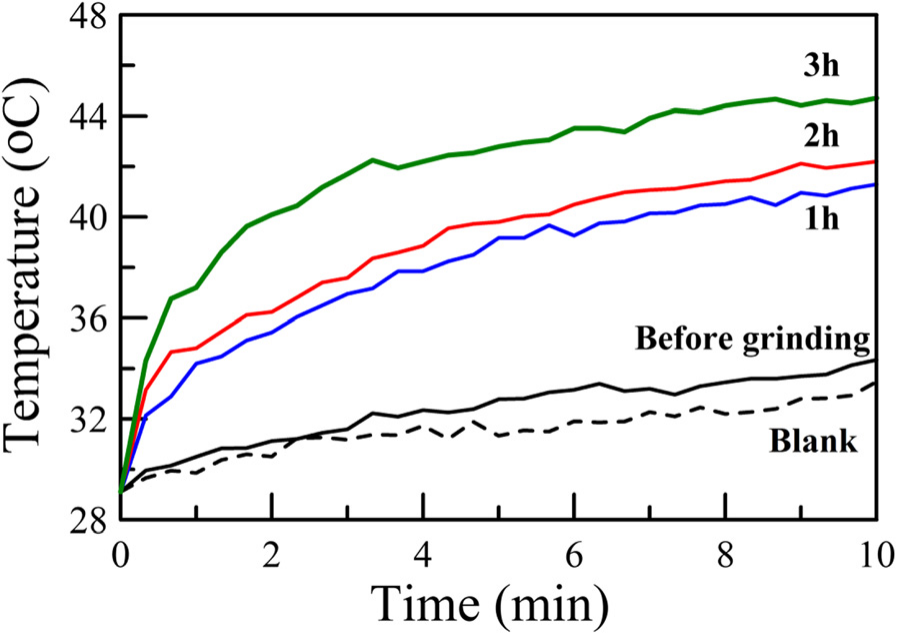
**Temperature variations for blank solution and aqueous dispersions of Cs**_**0.33**_**WO**_**3 **_**powder with NIR irradiation time.** The concentrations of Cs_0.33_WO_3_ powder before and after grinding for 1, 2, and 3 h were fixed at 0.008 wt.%. For the blank solution and the samples before grinding and after grinding for 1 and 2 h, 5 wt.% of PEG 6000 was added.

According to Figure 2, the mean hydrodynamic diameters of the Cs_0.33_WO_3_ powder before grinding and after grinding for 1, 2, and 3 h were 1,310, 250, 180, and 50 nm, respectively. Their NIR photothermal conversion property in the aqueous dispersions was examined at a fixed particle concentration of 0.008 wt.%. For the samples before grinding and after grinding for 1 and 2 h, 5 wt.% of PEG 6000 was added to avoid the occurrence of precipitation. The blank solution with 5 wt.% of PEG 6000 in deionized water was also investigated for comparison. The result was shown in Figure 8. It was obvious that, for the blank solution, the NIR irradiation (808 nm, 2.73 W/cm^2^) caused a temperature increase of only about 3°C after 10 min. For the aqueous dispersion of Cs_0.33_WO_3_ powder before grinding, the NIR irradiation-induced temperature increase was also slightly higher than the blank solution. However, for the aqueous dispersions of Cs_0.33_WO_3_ powder after grinding, the temperature was significantly raised under NIR irradiation. Also, with increasing grinding time, the temperature increase became more significant. For the aqueous dispersion of Cs_0.33_WO_3_ nanoparticles obtained after grinding for 3 h, the temperature increase after 10 min was 15°C. This was in agreement with the observation of absorption spectra and revealed that the NIR photothermal conversion capability of Cs_0.33_WO_3_ nanoparticles could be enhanced by the decrease of particle size.

**Figure 9:**
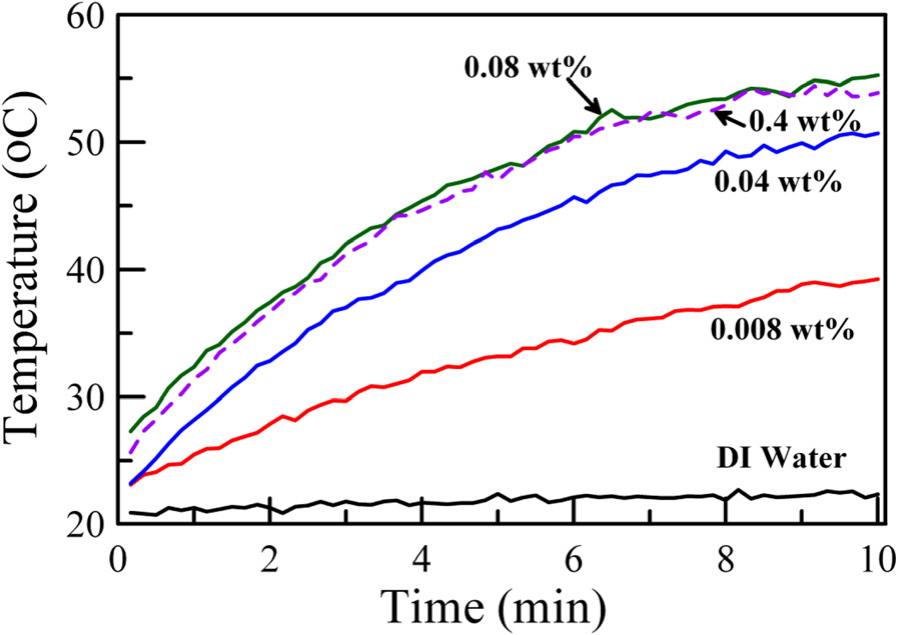
**Temperature variations for deionized water and aqueous dispersions of Cs**_**0.33**_**WO**_**3 **_**nanoparticles with NIR irradiation time**. Cs_0.33_WO_3_ nanoparticles were obtained after grinding for 3 h. Their concentrations ranged from 0.008 to 0.4 wt.%.

The variation of solution temperature with the NIR irradiation time for the aqueous dispersions of Cs_0.33_WO_3_ nanoparticles with different particle concentrations obtained after grinding for 3 h is shown in Figure 9, in which the result for deionized water was also indicated for comparison. It was obvious that the temperature increase owing to the photothermal conversion could be enhanced by increasing the particle concentration. When the concentration of Cs_0.33_WO_3_ nanoparticles was 0.08 wt.%, the solution temperature could be raised to about 55°C after 10 min. The temperature increase was above 30°C. This was consistent with the absorption spectra as indicated in Figure 7. However, when the concentration of Cs_0.33_WO_3_ nanoparticles was above 0.08 wt.%, the temperature increase could not be further enhanced. It was suggested that the absorption of NIR light by the Cs_0.33_WO_3_ nanoparticles might have reached the maximum, that is, the NIR light has been absorbed completely. This demonstrated that Cs_0.33_WO_3_ nanoparticles indeed possessed excellent NIR absorption and photothermal conversion property. Furthermore, the significant temperature increase of up to 55°C was sufficient for the killing of cancer cells [14,23]. Thus, in addition to NIR shielding, the other applications based on their excellent NIR photothermal conversion property (e.g., photothermal therapy) were expectable and worthy of further investigation.

According to the method reported by Chen et al. [35], the photothermal conversion efficiency for the aqueous dispersion of Cs_0.33_WO_3_ nanoparticles (2 mg/mL) under NIR irradiation (808 nm, 2.47 mW/cm^2^) could be determined to be 73%, close to that of gold nanorods with an effective radius of 30 nm. Because the Cs_0.33_WO_3_ nanoparticles examined had a mean hydrodynamic diameter of 50 nm and the photothermal conversion efficiency increased with the decrease of particle size [35], this result revealed that the resulting Cs_0.33_WO_3_ nanoparticles had a photothermal conversion property comparable to gold nanorods.

**Figure 10:**
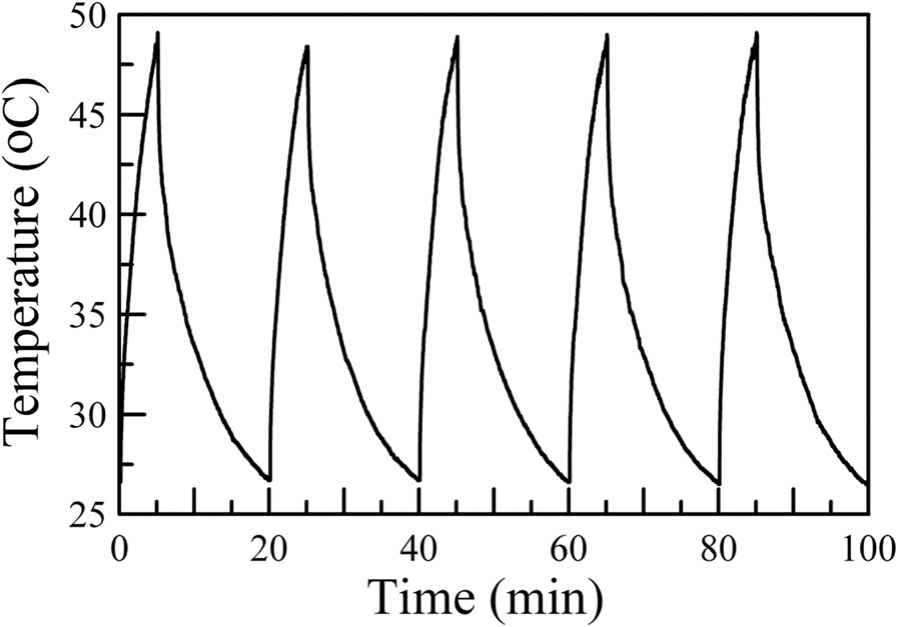
**Temperature variation for aqueous dispersions of Cs**_**0.33**_**WO**_**3 **_**nanoparticles with NIR irradiation time for 5 cycles.** Cs_0.33_WO_3_ nanoparticles were obtained after grinding for 3 h, and their concentration in the aqueous dispersions was 0.08 wt.%.

It was mentionable that recently, Fu et al. reported that the NIR irradiation by an 808-nm laser caused the partial melting of gold nanorods, leading to the decrease of photothermal conversion efficiency [36]. In this work, the photothermal stability of Cs_0.33_WO_3_ nanoparticles under the irradiation by an 808-nm diode laser was also examined. As shown in Figure 10, after 5 cycles, the Cs_0.33_WO_3_ nanoparticles had the same photothermal conversion capability. This revealed that Cs_0.33_WO_3_ nanoparticles possessed better photothermal stability than gold nanorods under NIR irradiation. Such an excellent property makes them to become a superior candidate in NIR photothermal therapy.

## Conclusions

Hexagonal Cs_0.33_WO_3_ nanoparticles with a mean hydrodynamic diameter of about 50 nm were prepared successfully in an aqueous solution of pH 8 by bead milling. They possessed excellent NIR photothermal conversion property and stability. With decreasing particle size or increasing particle concentration, the NIR photothermal conversion-induced temperature increase is enhanced. Such a nanomaterial not only could be used in the transparent solar heat-shielding filters, but also is useful for the development of NIR-triggered photothermal conversion materials in biomedicine.

## Competing interests

The authors declare that they have no competing interests.

## Authors’ contributions

CJC carried out the experiments and drafted the manuscript. DHC guided the study and modified the manuscript. Both authors read and approved the final manuscript.

## Authors’ information

CJC is currently a Ph.D. student of the National Cheng Kung University (Taiwan). DHC is a distinguished professor of the Chemical Engineering Department at National Cheng Kung University (Taiwan).
